# Comparative Genomics Analysis of *Streptomyces* Species Reveals Their Adaptation to the Marine Environment and Their Diversity at the Genomic Level

**DOI:** 10.3389/fmicb.2016.00998

**Published:** 2016-06-27

**Authors:** Xinpeng Tian, Zhewen Zhang, Tingting Yang, Meili Chen, Jie Li, Fei Chen, Jin Yang, Wenjie Li, Bing Zhang, Zhang Zhang, Jiayan Wu, Changsheng Zhang, Lijuan Long, Jingfa Xiao

**Affiliations:** ^1^Key Laboratory of Tropical Marine Bio-resources and Ecology and Guangdong Key Laboratory of Marine Materia Medica, South China Sea Institute of Oceanology – Chinese Academy of SciencesGuangzhou, China; ^2^Key Laboratory of Genome Sciences and Information, Beijing Institute of Genomics – Chinese Academy of SciencesBeijing, China; ^3^University of Chinese Academy of SciencesBeijing, China; ^4^Core Genomic Facility, Beijing Institute of Genomics – Chinese Academy of SciencesBeijing, China

**Keywords:** comparative genomics, streptomycete, pan-genomics, genome dynamics, marine environment adaptation

## Abstract

Over 200 genomes of streptomycete strains that were isolated from various environments are available from the NCBI. However, little is known about the characteristics that are linked to marine adaptation in marine-derived streptomycetes. The particularity and complexity of the marine environment suggest that marine streptomycetes are genetically diverse. Here, we sequenced nine strains from the *Streptomyces* genus that were isolated from different longitudes, latitudes, and depths of the South China Sea. Then we compared these strains to 22 NCBI downloaded streptomycete strains. Thirty-one streptomycete strains are clearly grouped into a marine-derived subgroup and multiple source subgroup-based phylogenetic tree. The phylogenetic analyses have revealed the dynamic process underlying streptomycete genome evolution, and lateral gene transfer is an important driving force during the process. Pan-genomics analyses have revealed that streptomycetes have an open pan-genome, which reflects the diversity of these streptomycetes and guarantees the species a quick and economical response to diverse environments. Functional and comparative genomics analyses indicate that the marine-derived streptomycetes subgroup possesses some common characteristics of marine adaptation. Our findings have expanded our knowledge of how ocean isolates of streptomycete strains adapt to marine environments. The availability of streptomycete genomes from the South China Sea will be beneficial for further analysis on marine streptomycetes and will enrich the South China Sea’s genetic data sources.

## Introduction

The specific environment conditions of the ocean, especially its high hydrostatic pressure, low temperature, and oligotrophic deep-sea (water depths greater than 1,000 m) conditions, have affected marine bacteria in various respects, forcing them to evolve special features to adapt to the unique deep-sea environment (Li et al., 2011; Qin et al., 2011, 2014). In general, the genomes of cultured deep-sea bacteria contain more transposable and phage-related elements and larger intergenic spacers than those of surface bacteria (Ivars-Martinez et al., 2008). Deep-sea bacteria also have more genomic islands than other bacteria that confer specific features, such as drug and heavy metal resistance (Qin et al., 2011). Moreover, genes that are important for cold, pressure, and oligotrophic adaptations, such as membrane unsaturation genes and signal transduction genes, are better represented in deep-sea bacteria than in other bacteria (Eloe et al., 2008; Qin et al., 2011). However, the genetic bases for marine adaptation are still unknown. Considering the high diversity of deep-sea bacteria (Qin et al., 2011), more genomic sequences of representative bacterial strains must be analyzed to characterize the mechanism of marine environmental adaptation.

Pan-genomics provide a new method for studying bacterial species diversity, evolution, adaptability, population structures and others (Medini et al., 2005; Muzzi et al., 2007; Zhang et al., 2011; Qin et al., 2014; Richards et al., 2014; Vernikos et al., 2015; Xiao et al., 2015). With the development of sequencing technology and sharp decreases in sequencing costs and time, more and more bacterial genomes are now openly available. This availability has made pan-genomics analysis possible for many strains at the species/genus level (Qin et al., 2014). A pan-genomic analysis of seven *Salmonella* strains provides insights into their genomic variations and clarifies their genomic evolution profile (Liang et al., 2012). A study on 11 marine *Glaciecola* strains revealed that the genus has an open pan-genome and contains some common genomic features related to cold adaptation, such as glycine betaine and exopolysaccharide synthesis genes as well as genes that encode cold-shock proteins and tRNA-dihydrouridine synthase (Qin et al., 2014). Therefore, with the accumulation of bacterial genomes, pan-genomics can be a very good method for exploring the phylogenetic relations and environmental adaptation mechanisms of bacterial genera.

Streptomycetes are a major branch of gram-positive bacteria with G+C contents generally ranging between 66 and 74% (Hopwood, 2006; Kämpfer, 2006). Most of these bacteria prefer neutral to alkaline soils as a natural habitat, and like other soil bacteria, they can also co-exist with earthworms or other arthropods within intestinal tracts or other body parts (Hulcr et al., 2011). Rivers, lakes, and marine environments, and especially the sediments of these environments, are also streptomycete habitats (Kämpfer, 2006). Although, streptomycetes are well-known for producing antibiotics that are used to treat bacterial, mycobacterial, fungal, and parasitic infections, some of them infect plants or humans and cause disease, such as potato scab and human mycetoma (Dunne et al., 1998; Loria, 2007). In comparison with streptomycetes from other environments, marine streptomycetes can produce a variety of novel active materials, and they have some different pathways (Li et al., 2011; Lee et al., 2014; Barakat and Beltagy, 2015) resulting from the uniquely high hydrostatic pressure, low temperature, and oligotrophy of the marine environment. However, the phylogenetic relations and genome diversity of streptomycetes between marine and other environments are still unknown.

In this study, we sequenced nine *Streptomyces* strains that were isolated from different longitudes, latitudes, and depths of the South China Sea. We compared them with the genomes of 22 NCBI downloaded streptomycete strains. The phylogenetic and pan-genomic analyses of the 31 strains reveal the evolutional relations of streptomycetes from different environments, and they also reveal streptomycete adaptation mechanisms to marine environments.

## Materials and Methods

### Bacterial Strains

Nine streptomycete strains (which belonged to four species, namely *S. abyssalis*, *S. oceani*, *S. qinglanensis*, and *S. nanshensis*) were isolated from different longitudes, latitudes, and depths of marine sediments or gorgonians in the South China Sea (detailed information is listed in Supplementary Table S1). Among these strains, *S. abyssalis* 10389, *S. abyssalis* 10390, and *S. nanshensis* 10429 were isolated from unidentified gorgonians, and others were separated from marine sediment. The sediment isolates were all taken from deep-sea environments (deeper than 500 m). Moreover, *Streptomycetes oceani* 02100 can survive only in medium that is prepared with seawater instead of distilled water, and the other strains can live with or without seawater.

Biomass was obtained by cultivating bacteria in modified ISP 2 broth (at 28°C, 1 week, 150 rpm). The genomic DNA extraction and purification of the representative strains were performed as described by Orsini and Romano-Spica (2001).

In addition, 22 genomes from *Streptomyces* genera were downloaded from the NCBI ftp (Bacteria and Bacteria_DRAFT parts in http://ftp.ncbi.nlm.nih.gov/genomes). They are all draft genomes except those of *S. fulvissimus* DSM 40593 and *S. griseus* NBRC 13350. These strains were separated from different habitats, such as soils, marine areas, plants, insect-associated substrate, humans, and so on (basic information for the 22 strains is listed in Supplementary Table S2).

### Genome Sequencing and Assembly

Paired-end and mate-pair libraries with insert sizes of 500 bp, 1–3 and 3–5 kb were constructed for nine streptomycete strains that were isolated from the South China Sea. All the libraries were sequenced using Illumina HiSeq2000 with a sequencing read length of 101 bp. The sequencing data were assembled with SOAPdenovo, version 2.04 (Luo et al., 2012). The total read lengths (after quality filtration) of each library, genome size, and average genome coverage of each strain are listed in Supplementary Table S3. The final genome assembly results are listed in Supplementary Table S4.

### Genome Annotation and Analysis

The tRNA genes were predicted by tRNAscan-SE (Lowe and Eddy, 1997). The rRNA genes were identified by RNAmmer (Lagesen et al., 2007), and the coding sequences (CDSs) were found with Prodigal, version 2.60 (Hyatt et al., 2010). The predicted genes were annotated by performing a BLAST (Altschul et al., 1990) search against databases of non-redundant proteins from the NCBI and COG (Tatusov et al., 2001; E-value 1e-5). Genes that were annotated as transporters and hypothetical proteins were selected, and they are regarded as transporter candidates. For these candidates, transporter predictions were made by performing a BLAST search against sequences that were downloaded from the TransportDB (Ren et al., 2007), which is widely used for comparative analyses of transport capabilities in prokaryotes (Ren and Paulsen, 2005, 2007). The metabolic pathways of streptomycetes were determined using the KEGG Automatic Annotation Server (KAAS; Moriya et al., 2007)^1^ with the bi-directional best hit (BBH) assignment method.

Moreover, the comparison of general genomic features, such as the genome size, G+C content and CDSs, were conducted with R. A principal component analysis (PCA) of these features was performed with R package psych (Revelle, 2015). Clustered regularly interspaced short palindromic repeats (CRISPRs) were found with pilercr1.06 (Edgar, 2007).

### Pan-genomics Analyses

All the genes from the 31 streptomycetes strains were delineated into clusters with putative shared homologies by MP method as implemented in the pan-genome analysis pipeline (PGAP) with a 50% cut-off for protein sequence identity. Pan-genome characteristic curves were depicted by PanGP (Zhao et al., 2014) with DG sampling algorithms. The COG functional enrichment of core genes was analyzed by PGAP with the parameter “–function.” Gene clusters that were shared among all strains and contained only single gene copies for each strain were called single copy core genes. The pairwise sequence alignment of all single copy core genes was performed with the BLAST program on the basis of an E-value cut-off of 1e-5.

### Single Copy Core Gene Identity Comparison

For this comparison, the single copy genes in the 31 streptomycete strains were first identified. The identities of each pair of genes between two independent strains were then determined by BLAST. A plot of the identities density of all pairs of genes between two independent strains was created with R.

### Phylogenetic Analyses

For the phylogenetic analysis, *Catenulispora acidiphila* DSM 44928 was selected as the outgroup. The gene clusters of the 32 strains were identified by PGAP using the MP method. The single copy core gene sequences from the 32 strains that belonged to one cluster were aligned with MAFFT (Katoh et al., 2002). The recombination for genes in these clusters was assessed using PhiPack [Bruen et al., 2006; which calculates the *p*-values for three individual methods, namely the neighbor similarity score (NSS), Maxχ^2^ and Phi] and GENECONV (Padidam et al., 1999). Recombination was inferred for *p*-values of less than 0.05. Thirty-three gene clusters (3.7%) that showed evidence of recombination for all four methods were removed according to the comparison analyzed by Richards et al. (2014). The rooted maximum likelihood (ML) species tree was constructed from a gene alignment concatenation of the 853 clusters with PhyML v3.0 (Guindon et al., 2010). The phylogenetic analysis was performed with the GTR + I + G substitution model, which was determined to have the best fit for the data when using jmodeltest-2.1.6 (Darriba et al., 2012). The rooted species tree based on 16S rRNA was also constructed with PhyML v3.0. For both phylogenetic trees, branch supports were provided by generating 100 bootstrap replicates.

### Gene Gain and Loss

The gene gain/loss on the species tree was assessed by parsimony-based gene-tree species-tree reconciliation as implemented in the AnGST program. The most parsimonious reconciliation was obtained by inferring a minimum set of evolutionary events [gene loss, gene duplication, speciation, lateral gene transfer (LGT), and gene birth or genesis] for a gene tree of every gene cluster containing three or more sequences, when using the default event penalty values of AnGST (LGT = 3.0, duplication = 2.0, loss = 1.0, and speciation = 0). We did not constrain the time-consistent reconciliation, allowing gene transfer events to occur between any two lineages.

The gene trees for all gene clusters containing three or more genes were constructed using PhyML v3.0 with the GTR + I + G substitution model. One hundred bootstrap replicates for every gene tree were provided to AnGST to account for phylogenetic uncertainty in the gene tree. Four gene clusters that contained particularly large number of sequences (more than 900) were excluded from the AnGST analysis. Genes in clusters containing only one gene were considered a birth in every genome. For gene clusters containing two genes, we explained the evolutionary history of these genes by following the method mentioned by Richards et al. (2014).

## Results

### General Genomic Information on Streptomycetes

Constructing genomic maps of streptomycetes is a great challenge as a result of the high GC contents, linear chromosomes, unstable chromosome structures and large genome sizes of these bacteria. In this study, libraries with various insert fragment lengths were used for genome sequencing and assembly, and the sequencing depths of these nine strains were all approximately 300×. We used two software of Velvet (Zerbino and Birney, 2008) and SOAPdenovo2 (Luo et al., 2012) to obtain optimal assembly results. After the comparison of the N50 scaffolds and Contigs from two software, we chose SOAPdenovo2 to conduct the genome assembly. The scaffold numbers ranged from 22 to 2,310. The scaffold N50 were all above 14 kb (Supplementary Table S4).

**FIGURE 1:**
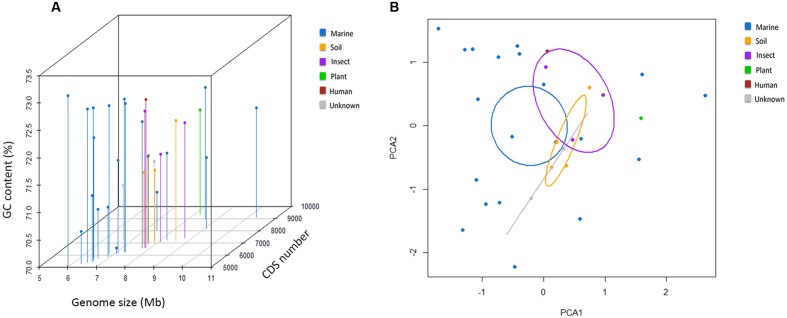
**General genomic features of 31 streptomycete strains. (A)** Three-dimensional plots of the GC content, genome size and CDS number. **(B)** The principal component analysis (PCA) of the genome size, CDS number and G+C content.

The general genome features of the nine newly sequenced streptomycete strains and 22 previously sequenced streptomycete strains are summarized in **Table 1**. Twenty-two previously sequenced streptomycete strains were selected through the phylogenetic analyses of 136 streptomycete strain genomes (127 strain genomes were downloaded from NCBI and nine strain genomes were sequenced in this study) with Co-phylog (Yi and Jin, 2013; Supplementary Figure S1). The G+C contents of 31 streptomycete strains were all above 70% (70.1–73.1%). The genomes of *S. fulvissimus* DSM 40593 and *S. griseus* NBRC 13350 were 7.91 and 8.55 Mb in size, respectively. The others were draft genomes with sizes ranging from 5.93 to 10.27 Mb. The predicted protein CDSs ranged from 5,163 to 9,122. Here, we could conclude that the streptomycete genome size and CDSs number have great variability. The CDSs number was usually consistent with the genome size (*R* = 0.84; **Figure 1A**). Most streptomycete strains derived from marine environments had smaller genome sizes, but more than half of them (11/20, and 20 strains are isolated from marine environments) possessed a slightly higher GC content (**Figure 1A**). *Streptomyces* sp. CNS606 had the smallest genome size (5.93 Mb) but the highest G+C content (73.1%; **Figure 1A**). We also tested the continuous distributions of these three general features in marine-derived streptomycete strains and strains from other sources by two-tailed Kolmogorov–Smirnov test. The CDSs numbers and genome sizes had significantly different continuous distributions (with *p*-values of 0.003 and 0.006, respectively). However, the continuous distributions of the G+C content were not remarkable (*p*-value 0.676). According to the PCA, the genome size, CDS number and G+C content were divided into two principal components (**Figure 1B**). The majority of the marine-derived streptomycete strains can be divided from other sourced strains by the first dimension (genome size and CDS number), with ellipses on 95% confidence intervals of environment classifications (PCA1 61% variance, PCA2 34% variance).

**Table 1:** Genomic information of 31 *Streptomyces*.

Strains	Source	Size (Mb)	GC (%)	CDS no.	Average CDS length (bp)	tRNA no.	rRNA no.	Study
*S. abyssalis* 10389	Gorgonian	6.91	70.9	5884	980	57	14	This study
*S. abyssalis* 10390	Gorgonian	6.61	70.9	5724	1009	54	10	This study
*S. nanshensis* 10429	Gorgonian	7.73	71.7	6941	965	59	3	This study
*S. nanshensis* 01066	Marine sediment	7.43	70.8	8047	794	52	5	This study
*S. oceani* 02100	Marine sediment	6.33	70.6	5274	1047	56	3	This study
*S. nanshensis* 10372	Marine sediment	10.27	72	9122	923	75	8	This study
*S. nanshensis* 10374	Marine sediment	6.47	72.8	5402	1034	60	13	This study
*S. qinglanensis* 10379	Marine sediment	6.61	72.8	5506	1037	58	13	This study
*S. nanshensis* 10399	Marine sediment	8.58	72.4	8985	680	69	2	This study
*S. sulphureus* DSM 40104	Marine	7.1	71.7	6173	996	58	12	-
*S. sulphureus* L180	Marine sediment	6.47	72.2	5786	969	47	2	Zhao X.Q. et al., 2012
*Streptomyces* sp. AA1529	Marine sediment	7.29	72.7	6303	1008	60	6	Xiong and Wang, 2012
*Streptomyces* sp. W007	Marine sediment	9.06	71.3	8197	979	64	4	-
*Streptomyces* sp. CNB091	Marine	8.23	71.6	7216	1003	69	9	-
*Streptomyces* sp. CNH287	Marine	6.79	72.7	6163	970	59	14	-
*Streptomyces* sp. CNS606	Marine	5.93	73.1	5163	1022	60	11	-
*Streptomyces* sp. CNT318	Marine	7.28	72.8	6255	1015	63	18	-
*Streptomyces* sp. CNT360	Marine	7.71	72.3	6613	1017	58	15	-
*Streptomyces* sp. TAA204	Marine	6.58	71.2	5504	1050	59	14	-
*Streptomyces* sp. TAA486	Marine	7.07	70.1	6148	1000	56	15	-
*S. globisporus* C-1027	Soil	7.69	71.6	7019	972	57	1	Wang et al., 2012
*S. griseus* NBRC 13350	Soil	8.55	72.2	7183	1053	66	18	Ohnishi et al., 2008
*S. roseosporus* NRRL 11379	Soil	7.85	71.3	7129	966	66	9	Miao et al., 2005
*S. roseosporus* NRRL 15998	Soil	7.56	71.3	6958	961	66	9	Liu et al., 2011
*Streptomyces* sp. CcalMP-8W	Insect-associated	7.81	72.5	6581	1046	63	7	-
*S. griseus* XylebKG-1	Ambrosia beetle	8.73	72.1	7436	1037	66	18	Grubbs et al., 2011
*Streptomyces* sp. HPH0547	*Homo sapiens*	7.82	72.7	6641	1023	57	17	-
*Streptomyces* sp. ScaeMP-e10	Insect-associated	8.07	71.6	7119	1015	67	11	-
*Streptomyces* sp. Wigar10	Surface-sterilized garlic bulb	8.18	71.9	9365	750	61	4	Klassen et al., 2011
*S. fulvissimus* DSM 40593	Unknown	7.91	71.5	6984	1009	73	18	-
*Streptomyces* sp. HCCB10043	Unknown	6.69	71	7236	825	51	2	Rao et al., 2011

### Phylogenetic Analyses of Streptomycetes

**FIGURE 2:**
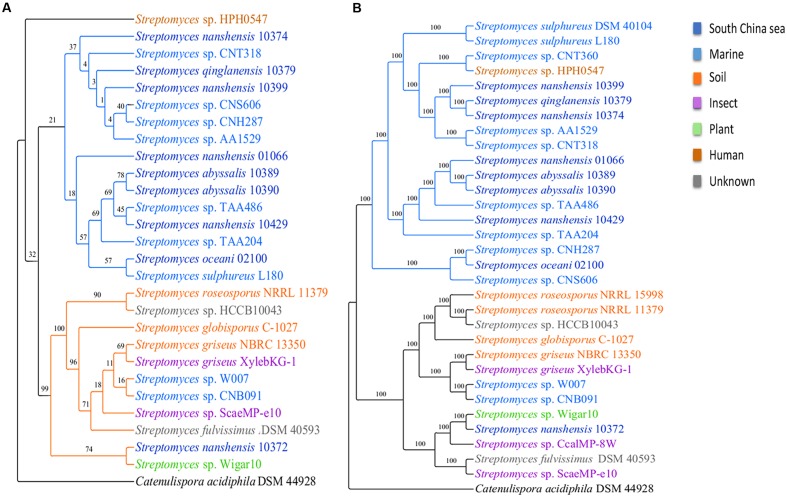
**Phylogenetic trees for streptomycete strains.** The phylogenetic trees were inferred using maximum likelihood on the basis of **(A)** the 16S rRNA gene and **(B)** the concatenated alignment of single copy core genes. The numbers on the branches represent the support derived from 100 bootstrap replicates.

To infer the phylogenetic relations of 31 streptomycete strains, we reconstructed phylogenetic trees on the basis of 16S rRNA and single copy core genes with *Catenulispora acidiphila* DSM 44928 as the outgroup (for details, see Materials and Methods). In general, the two trees shared the same topology. In the phylogenetic tree based on 16S rRNA gene sequences (**Figure 2A**), 27 strains were primarily grouped into two subgroups, with the exception of *Streptomyces* sp. HPH0547. The *S. sulphureus* DSM 40104, *Streptomyces* sp. CNT360, *S. roseosporus* NRRL 15998 and *Streptomyces* sp. CcalMP-8W strains were not included in this tree because 16S rRNA genes were not detected in their draft genomes. However, the internal branches of the tree that were based on 16S rRNA gene sequences had low bootstrap support. Single-gene phylogenies might not always reflect the evolutionary history of a species due to the high degree of LGT (Marri et al., 2006). We also reconstructed a phylogenetic tree using concatenated single copy core genes (**Figure 2B**). This tree exhibited larger bootstrap scores and higher robustness. According to the tree based on concatenated single copy core genes, 31 streptomycete strains were clearly grouped into two subgroups. One subgroup with 18 strains contains 17 strains isolated from marine environments, and one from human beings is called the marine-derived subgroup. The other subgroup contains 13 strains that were isolated from different environments, such as marine areas, soil, insects, plants, and others, and it is called the multiple sources subgroup. The tree of concatenated single copy core genes suggested that *S. nanshensis* 10399, *S. qinglanensis* 10379 and *S. nanshensis* 10374 are tightly grouped and that *S. abyssalis* 10389 and *S. abyssalis* 10390 are sister taxa that are equally closely related to *S. nanshensis* 01066. This finding may reflect that close, isolated locations are associated with genetic recombination more frequently, which may reduce the divergence of these bacteria. Our analysis suggests that *Streptomyces* sp. CNH287, *S. oceani* 02100 and *Streptomyces* sp. CNS606 form a separate clade. *Streptomyces* sp. HPH0547 was isolated from *Homo sapiens*, and it was clustered in the marine-derived group as an exception. This finding may imply that this strain originated in the ocean and that its ancestor was transmitted to humans because of frequent activity. The three marine-derived strains (*Streptomyces* sp. W007, *Streptomyces* sp. CNB091, and *S. nanshensis* 10372) were clustered into a multiple sources subgroup with some soil-derived strains. We boldly conjecture that these three bacterial ancestors were transmitted to the marine environment recently, and they still retain many characteristics from the land.

Tang et al. (2013) found sharp genetic distinctions among bacteria from closely related lineages. They showed the existence of an abrupt turning point in the sequence divergence between any pair of *Salmonella* lineages in their comparisons (Tang et al., 2013). We compared the sequence identity levels between the single copy core genes among 31 strains. In the marine-derived subgroup, most of the genes shared 85% of their sequence identity. Because *S. nanshensis* 10374 and *S. qinglanensis* 10379, *S. abyssalis* 10389 and *S. abyssalis* 10390 exhibited high similarity, there is a small peak at the 100% sequence identity point (Supplementary Figure S2A). In the multiple sources subgroup, the majority of sequence identities among the strains were above 90%, and the peak of the identity distribution was at 95%. Similarly, we also calculated the percentages of genes with 80% or higher shared identity (Supplementary Figure S2B), and more than 90% (Supplementary Figure S2C) of the identities between each pair of genes were found in the single copy core genes of 31 strains. In general, these results suggested that the genomic conservation that presented within the multiple sources subgroup is relatively high.

Recombination plays a key role in bacterial adaptation. We also estimated the relative effects of recombination and mutation (r/m) for each strain through the concatenated alignment of all single copy orthologs with ClonalFrameML (Didelot and Wilson, 2015). The r/m ratio for each branch in the tree ranged from 0.23 on the branch to *S. griseus* XylebKG-1 to 116.45 on the branch to *S. oceani* 02100 (Supplementary Table S5). The high r/m ratio of *S. oceani* 02100 suggested that the overall recombination caused 116 times more substitutions than mutations, confirming the importance of recombination in helping *S. oceani* 02100 adapt to the variable ocean environment. However, *S. griseus* XylebKG-1 is an ambrosia beetle-associated actinomycete, and its low r/m ratio suggests long-term co-evolution with its host. The r/m ratios in the marine-derived subgroup were clearly different from those in the multiple sources subgroup (Welch’s two sample *t*-test, *p*-values 0.024). This finding showed that most marine-derived streptomycetes had obviously higher r/m ratio than those multiple sources streptomycetes.

### Streptomycete Genome Dynamics

**FIGURE 3:**
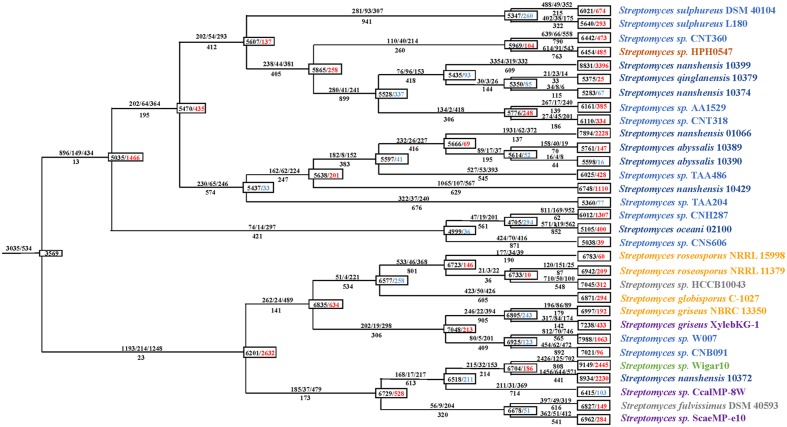
**Gene gain and loss events during the evolution of 31 streptomycete strains.** The numbers in boxes on every node represent the genome size and the overall balance (gain-loss: balance), with black indicating the genome size, red indicating an overall gain and blue indicating an overall loss. The numbers above and below every lineage show the number of birth/duplication/transfer and loss events.

To clarify the genome dynamics of streptomycetes, we analyzed the gene gain/loss events that occurred during the evolution of every strain using AnGST (David and Alm, 2011). We identified 29,695 clusters of functional genes containing a total of 210,057 gene copies with using MP method in PGAP (Zhao Y.B. et al., 2012). Among these clusters, 9,293 (31.3%) possessed three or more gene copies, 2,562 (8.6%) included two gene copies (doublets), and 17,840 (60.1%) contained one gene copy (singletons). Using the default penalties, we detected a dynamic pattern of gene gain/loss through the phylogeny. During the process of forming the two subgroups, namely the marine-derived subgroup and the multiple sources subgroup, more (1,466 and 2,632, respectively) gene gain events occurred (**Figure 3**). During this period, the multiple sources subgroup had more than 79.5% of the genes in comparison with the marine-derived subgroup, which always had a larger genome. Subsequently, some branches of the two subgroups excluding the terminal branches experienced several periods of genome shrinkage (more gene loss than gain). Finally, in recent times, nearly all 31 (27/31) of the strains except for *S. nanshensis* 10374, *S. abyssalis* 10390, *Streptomyces* sp. TAA204, and *Streptomyces* sp. CcalMP-8W were characterized by much more gains than losses. Phylogenetic analyses of 46 *Streptococcus* strains by Richards et al. (2014) acquired a similar pattern of genome evolution. Of the proportion of genes gained on the terminal branches, 30.7% were identified as LGT. The majority (60.1%) of the acquired genes were classified as genes that were born on these branches (singletons). However, given that these genes were not present in any other strains, it is possible that they were acquired via LGT from bacteria that were not included in this study. To explore this possibility, each singleton gene was searched for significant sequence matches using BLASTp (E-value cutoff = 1e-5) against the NCBI NR database. A total of 70.2% (12,531) of the singleton genes matched other species, which indicated that over two-thirds of the singletons were actually acquired through LGT. In addition, 94.2% (11,810) of these genes match a streptomycete species. Lefebure et al. (2012) performed a similar AnGST analysis on 15 *Streptococcus* species, and they showed that these genes account for approximately two-thirds of the genes born on the *Streptococcus pyogenes* branch. Although, the remaining singleton genes may be born *de novo* on this branch, it is also possible that homologous genes haven’t yet to be sequenced (Richards et al., 2014). Overall, our findings suggest that a large proportion of the streptomycete pan-genome (all gene clusters) has been involved in LGT. Among all the gene clusters, 60.2% (17,876) were identified as being involved in LGT.

From the perspective of gene gain/loss for the phylogenetic process of individual species, *S. oceani* 02100 and *S. nanshensis* 10372 are notable. After the initial expansion period (with a gain of 1,466 genes), *S. oceani* 02100 underwent two consecutive periods of gene loss (it lost 330 genes in total) and recently obtained 400 genes. In comparison with other strains, the *S. oceani* 02100 strain obtained fewer genes during evolution, which may have caused it to be unable to live without salt (Tian et al., 2012). *S. nanshensis* 10372 experienced only one period of streamlining (lost 211 genes) during evolution and recently gained 2,230 genes. These changes all resulted in *S. nanshensis* 10372s possession of the largest genome size among the nine marine strains.

### Core and Pan-Genome Analyses

**FIGURE 4:**
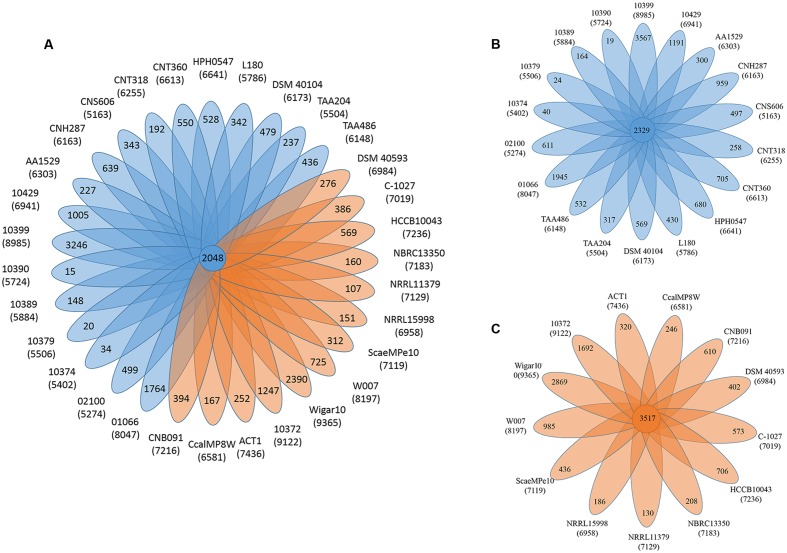
**The pan-genome of streptomycetes. (A)** Flower plots showing the core gene number (in the center) and strain-specific gene number (in the petals) in 31 streptomycete strains. **(B)** Flower plots showing the core gene number (in the center) and strain-specific gene number (in the petals) in the marine-derived subgroup. **(C)** Flower plots showing the core gene number (in the center) and strain-specific gene number (in the petals) in the multiple sources subgroup. The numbers under the strain name represent the total number of coding proteins. Blue indicates the marine-derived subgroup and orange indicates the multiple sources subgroup.

**FIGURE 5:**
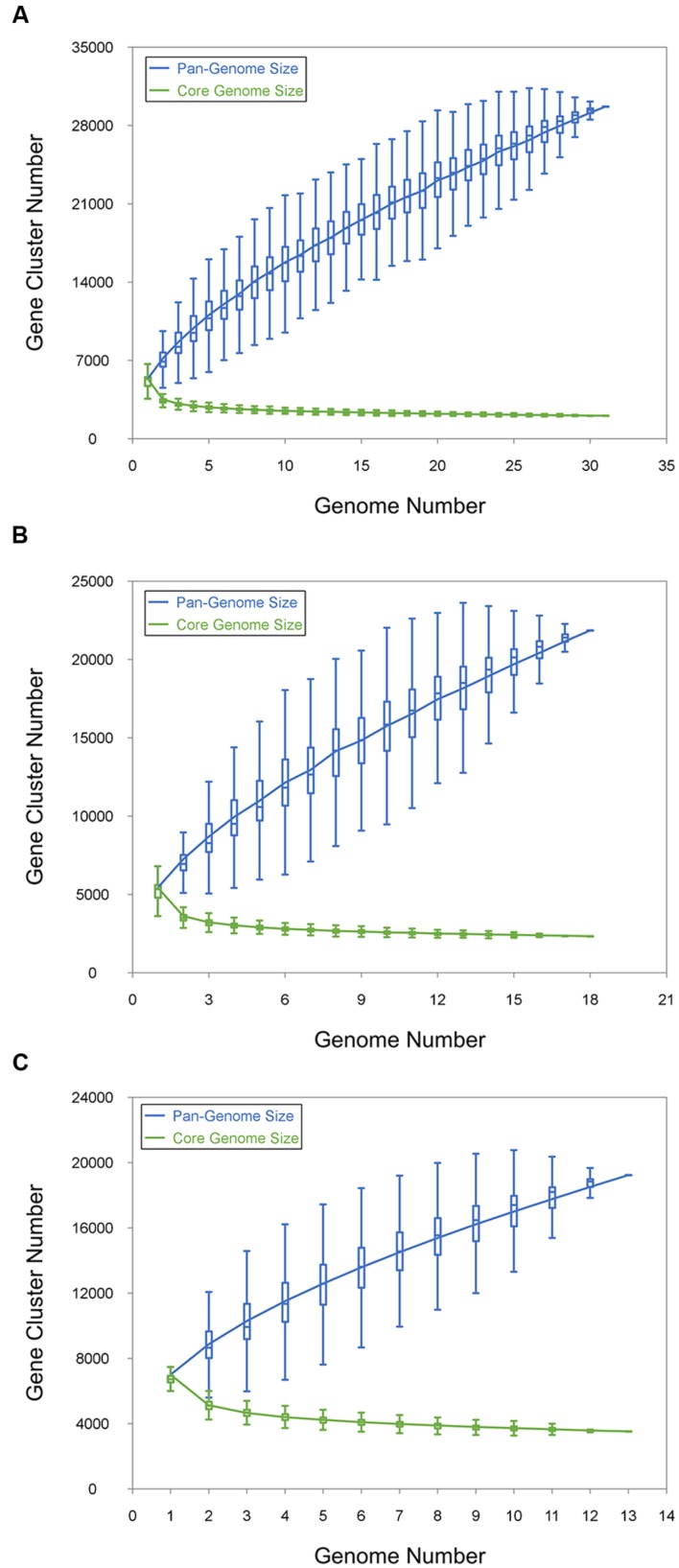
**Pan- and core genome size prediction. (A)** Curves for 31 streptomycete pan-genomes and core genomes. **(B)** Curves for the pan-genomes and core genomes of 18 marine-derived strains. **(C)** Curves for the pan-genomes and core genomes of 13 multiple source strains. The blue dots denote the pan-genome size and the green dots denote the core genome size. The connected median values represent the relations between the genome number and gene cluster number.

**FIGURE 6:**
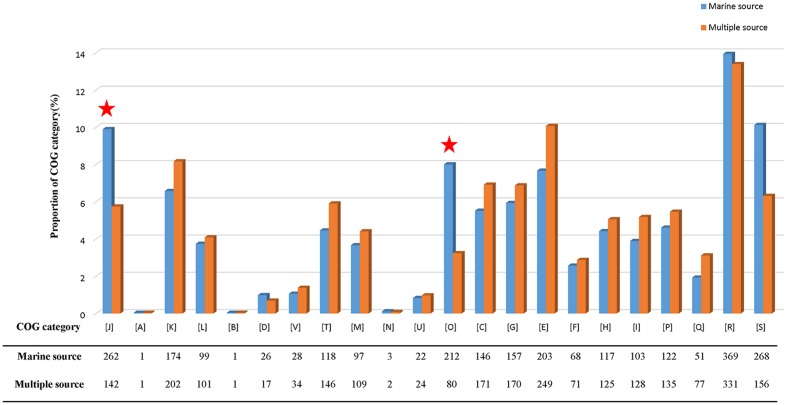
**COG category percentage of core genes in the marine-derived subgroup and multiple sources subgroup.** The COG categories are described as follows: J, translation, ribosomal structure, and biogenesis; A, RNA processing and modification; K, transcription; L, replication, recombination, and repair; B, chromatin structure and dynamics; D, cell cycle control, cell division, and chromosome partitioning; V, defense mechanisms; T, signal transduction mechanisms; M, cell wall/membrane/envelope biogenesis; N, cell motility; U, intracellular trafficking, secretion, and vesicular transport; O, post-translational modification, protein turnover, and chaperones; C, energy production and conversion; G, carbohydrate transport and metabolism; E, amino acid transport and metabolism; F, nucleotide transport and metabolism; H, coenzyme transport and metabolism; I, lipid transport and metabolism; P, inorganic ion transport and metabolism; Q, secondary metabolites biosynthesis, transport and catabolism; R, general function prediction only; and S, function unknown.

The pan-genome defines the entire genomic repertoire of a given phylogenetic clade and encodes for all possible lifestyles of its organisms (Vernikos et al., 2015). This genome primarily contains the core genome, dispensable genome and strain-specific genes. The core genome is essential for a bacterium basic lifestyle, and the dispensable genome offers species diversity, environmental adaptation and other characteristics (Tettelin et al., 2008). To understand the genetic composition of the 31 streptomycete strains in the pan-genome insight more thoroughly, we clustered all 210,057 protein CDSs in 31 streptomycete strains with PGAP. All the protein CDSs were clustered into 29,695 orthologs; 2,048 (6.90%) orthologs were identified in 31 strains as the streptomycete core genome (**Figure 4A**), and 1,272 of them were single-copy. The 9,807 orthologs were identified as dispensable genomes, and 17,840 genes were strain-specific. *S. nanshensis* 10374 and *S. qinglanensis* 10379 as well as *S. abyssalis* 10389 and *S. abyssalis* 10390 had a smaller number of strain-specific genes because these two pairs of strains had high similarities in the genome. However, *S. nanshensis* 10399 had the largest number of strain-specific genes, indicating that it has the greatest difference with the other strain genomes. For further analysis, we also identified the core ortholog genes and strain-specific genes in marine-derived and multiple source subgroups (**Figures 4B,C**). In an attempt to understand the relations between the streptomycete pan-genome size, the core genome number and the strain number, we plotted the pan-genome profile fitted curves of the 31 strains, the marine-derived subgroup and the multiple sources subgroup according to a Heaps’ law model (Tettelin et al., 2005, 2008) with PanGP (Zhao et al., 2014). As shown in **Figure 5A**, we can intuitively observe that the more genomes we added, the more new ortholog clusters were discovered, implying an open pan-genome of streptomycete strains (**Figure 5A**, blue curve). The green curve in the same picture, which is fit by an exponential function, indicated that the average number of core genes converged to a relatively constant number of 2,266 (2264–2268, 95% confidence interval). In the marine-derived subgroup and the multiple sources subgroup, the pan-genome profiles were basically similar (**Figures 5B,C**). We have also conducted a COG function category comparison among core genes from the marine-derived subgroup and the multiple sources subgroup. As shown in **Figure 6**, the core genomes of these two subgroups both harbor a large proportion of genes pertaining to transcription (K), replication, recombination and repair (L), signal transduction mechanisms (T), cell wall/membrane/envelope biogenesis (M), energy production and conversion (C), carbohydrate transport and metabolism (G), amino acid transport and metabolism (E) and others. The marine-derived subgroup contains a higher proportion of core genes belonging to the COG categories of translation, ribosomal structure and biogenesis (J), and post-translational modification, protein turnover, and chaperones (O). The abundance of these genes might play a role in bacterial adaptations to the low temperature, high pressure, oligotrophic, saline and dark marine environment by ensuring protein synthesis and maintaining protein functional stability. The overrepresentation of COG category J was also observed in the genome of the halophilic archaeon *Halococcus hamelinensis* (Gudhka et al., 2015). The abundance of COG category O was also consistent with previous research (Kuwahara et al., 2007; Wang et al., 2008).

### Transporters and Various Marine Environment Adaptations

**FIGURE 7:**
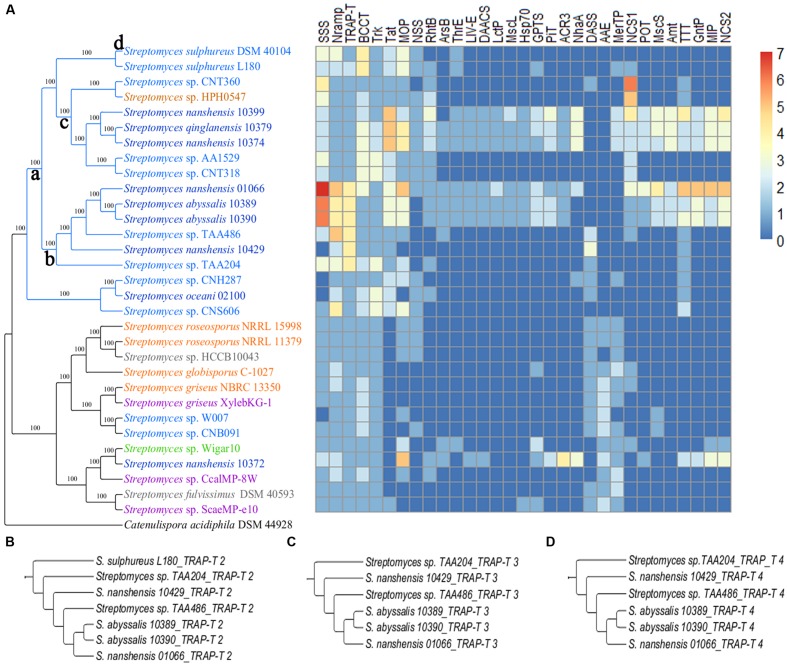
**(A)** A streptomycete species tree and a heatmap showing the distribution of transport protein families. The transport protein families are described as follows: SSS, solute: sodium symporter; Nramp, metal ion (Mn^2+^-iron) transporter; TRAP-T, tripartite ATP-independent periplasmic transporter; BCCT, betaine/carnitine/choline transporter; Trk, K^+^ transporter; Tat, twin arginine targeting; MOP, multidrug/oligosaccharidyl-lipid/polysaccharide; NSS, neurotransmitter: sodium symporter; RhtB, resistance to homoserine/threonine; ArsB, arsenite-antimonite; ThrE, threonine/serine exporter; LIV-E, branched chain amino acid exporter; DAACS, dicarboxylate/amino acid: cation (Na^+^ or H^+^) symporter; LctP, lactate permease; MscL, large conductance mechanosensitive ion channel; Hsp70, cation channel-forming heat shock protein-70; GPTS, general phosphotransferase system; PiT, the inorganic phosphate transporter; ACR3, arsenical resistance-3; NhaA, Na^+^: H^+^ antiporter; DASS, divalent anion: Na^+^ symporter; AAE, aspartate: alanine exchanger; MerTP, mercuric ion (Hg^2+^) permease; NCS1, nucleobase: cation symporter-1; POT, proton-dependent oligopeptide transporter; MscS, small conductance mechanosensitive ion channel; Amt, ammonia transporter channel; TTT, tricarboxylate transporter; GntP, gluconate: H^+^ symporter; MIP, the major intrinsic protein; and NCS2, the nucleobase:cation symporter-2. **(B)** The gene tree of TRAP-T 2. **(C)** The gene tree of TRAP-T 3. **(D)** The gene tree of TRAP-T 4.

To investigate how streptomycetes interact with environments, we compared how cytoplasmic transport systems differ between the two subgroups. All the strains in both subgroups contain a large number of ATP-binding cassette transporters (ABC transporters) and the major facilitator superfamily (MFS). The transporters in these two families are ubiquitously present in all biological kingdoms. More transporters, such as the solute: sodium symporter (SSS), metal ion (Mn^2+^) transporter (Nramp), tripartite ATP-independent periplasmic transporters (TRAP-T) and others are found among the strains in the marine-derived subgroup that showed a significant difference (**Figure 7A**). Some transporters occur specifically in strains from the South China Sea, such as the branched chain amino acid exporter (LIV-E), small conductance mechanosensitive ion channel (MscS) and large conductance mechanosensitive ion channel (MscL; **Figure 7A**). During the transporter analysis, we found that marine-derived streptomycete strains enriched the K^+^ transporter (Trk) and (betaine/carnitine/choline transporter) BCCT more than multiple sources did (Supplementary Table S6). The enrichment of Trk may help marine-derived streptomycete strains accumulate K^+^, which is the primary strategy for many extremophiles that survive in high-osmolality environments (Roberts, 2004). An abundance of BCCT was also observed in several marine *Vibrio* strains, such as *V. vulnificus* and *V. fischeri* (Naughton et al., 2009). BCCT would assist in the accumulation of betaine, carnitine and choline, which are known as compatible solutes. These compatible solutes not only maintain the cellular osmotic balance but also serve as stabilizers of proteins and cell components against the denaturing effects of high ionic strength (Kempf and Bremer, 1998). The transporter analysis also showed that most streptomycete strains that are derived from the South China Sea possess MscS and MscL proteins, except *S. oceani* 02100, *S. nanshensis* 10372 and *S. nanshensis* 10429. When bacteria are moved from marine to freshwater environments (leading to osmotic downshock), the mechanosensitive channels may help the cells reduce their turgor pressure rapidly by releasing cytoplasmic solutes (Berrier et al., 1992). Consistent with previous reports, the MscL protein alleviated cell lysis following a sudden osmotic downshock in the marine halophile *Vibrio alginolyticus* (Nakamaru et al., 1999).

Strains of the marine-derived subgroup contained more transporters that were involved in nutrient absorption, such as TRAP-T, SSS, the nucleobase: cation symporter-1 (NSC1), the twin arginine targeting (Tat) family and the neurotransmitter: sodium symporter (NSS). Substrate binding proteins (SBPs) that are anchored in the cytomembrane of gram-positive bacteria have high affinities to the substrate and assist bacteria in taking advantage of low nutrient concentrations. The known SBP-dependent secondary transporters are currently categorized into two families, namely TRAP-T and the tripartite tricarboxylate transporters (TTTs; Mulligan et al., 2011). Although, there is no obvious sequence consistency between TRAP-T, SSS and NSC1, the latter two have the same folding structure and the same transport mechanism as the TRAP-T (Weyand et al., 2008). The Tat family is likely to play a major role in nutrient acquisition from complex sources, permitting streptomycetes growth when more readily used soluble nutrients are not available. The known or predicted Tat substrates in *S. coelicolor* include a diverse array of hydrolytic enzymes (Chater et al., 2010). Moreover, a comparison of the two-component system indicates that 15 strains of the marine-derived subgroup can assimilate phosphate under phosphate-limiting conditions, and only four strains in the other subgroup have the effective pathway. Furthermore, according to the AnGST results, the gene-tree species-tree reconciliation allows us to infer the evolutionary history of every gene family in an explicit fashion. For the example of TRAP-T, there are four clusters of TRAP-T genes in the data set. The first TRAP-T cluster contained 31 genes, with one for each strain. This cluster was born on the root (**Figure 7A**) and has been retained throughout evolutionary history. This finding suggests that the TRAP-T 1 gene (TRAP transporter solute receptor, TAXI family) is important to *Streptomyces* strains. The other three cluster gene trees were shown in **Figures 7B–D**. For TRAP-T 2 (permease with DctM domain; **Figure 7B**), it first gained on branch a and then lost on branch c and branch d. For TRAP-T 3 (hypothetical protein with DctQ domain; **Figure 7C**) and TRAP-T 4 (hypothetical protein with PBP2_TRAP_Siap_TeaA_like domain; **Figure 7D**), they were all born on branch b. Not surprisingly, *S. nanshensis* 01066, *S. abyssalis* 10389, *S. abyssalis* 10390, *Streptomyces* sp. TAA486, *S. nanshensis* 10429, and *Streptomyces* sp. TAA204 had three protein components of TRAP-T (TRAP-T 1, TRAP-2, and TRAP-T 3), which helps these strains cope with the oligotrophic marine environment.

Most strains that were isolated from the South China Sea also have other transporter families that are involved in taking up nutrients, such as the TTT, the gluconate: H^+^ symporter (GntP), the dicarboxylate/amino acid: cation (Na^+^ or H^+^) symporter (DAACS), the lactate permease (LctP), proton-dependent oligopeptide transporter (POT), the general phosphotransferase system (GPTS) and the ammonia transporter channel (Amt). As mentioned above, the TTT permits streptomycetes to take up nutrients under oligotrophic conditions. The remaining families enable streptomycetes to make use of alternative carbon sources and nitrogen sources and to obtain more energy and thereby increase their competitiveness in response to ecological stress (Kleiner, 1985; Caspari and Urlinger, 1996; Yurgel et al., 2000; Nunez et al., 2002; Govindarajan et al., 2013; Lyons et al., 2014). Additionally, the PhoR-PhoB two-component system and the multiple sugar ABC transporter that are contained in more strains of the marine-derived subgroup may also help these strains take full advantage of nutrients under oligotrophic conditions. The PhoR-PhoB two-component system plays an important role in detecting and responding to changes in the environmental phosphate concentration (Marzan and Shimizu, 2011). Thus, the system facilitates the ability of the marine-derived subgroup strain to regulate its phosphate uptake in a sophisticated manner and survive, even under phosphate-limiting conditions. Most marine-derived strains possessed a complete, putative multiple sugar transport system (ChvE, GguB, GguA). ChvE has been suggested to be involved in sugar binding, sugar utilization and virulence in *Agrobacterium tumefaciens* (He et al., 2009). Previous research has also indicated that GguA and GguB play a role in sugar utilization. In *A. tumefaciens*, the chvE-, gguA-, and gguB-encoded products are predicted to constitute a complete binding protein-dependent ABC transporter, which has a wide range of substrates, including L-arabinose, D-fucose, D-galactose, D-glucose, and D-xylose (Zhao and Binns, 2011).

In addition to the above transporter families, strains from the marine-derived subgroup also possess transporters that participate in drug/metabolite transport, metalloid transport, and pH regulation. Members pertaining to resistance-nodulation-cell division (RND), the drug/metabolite transporter (DMT), multidrug/oligosaccharidyl-lipid/polysaccharides (MOPs), and resistance to homoserine/threonine (RhtB) are involved in drug/metabolite or homoserine lactone efflux (Jack et al., 2001; Li et al., 2011), and there are more strains from the marine-derived subgroup than from the multiple sources subgroup. Members of the threonine/serine exporter (ThrE) family contained more strains that were isolated from the South China Sea than other strains. Threonine and serine will be exported when their high concentrations inhibit cell growth (Yen et al., 2002). The proper content of manganese (Mn), which is called a “life guard,” is important for maintaining the normal physiological function of the cell (Li and Tang, 2012). The presence of more members of the metal ion (Mn^2+^) transporter (Nramp) family in the marine-derived subgroup strains reflects their adaptability to low marine Mn^2+^ concentrations (Komorsky-Lovric, 1998; Yen et al., 2002). Members of the arsenical resistance-3 (ACR3) and arsenite-antimonite (ArsB) efflux family included more strains that were isolated from the South China Sea, and these proteins pumped As(III) and Sb(III) away to reduce toxicity and enhance survivability (Rosen, 1996; Ostrowski et al., 1999; Hughes, 2002; Fu et al., 2009). Additionally, these strains have more Na^+^: H^+^ antiporters (NhaA), which are involved in maintaining the pH homeostasis and exhausting the cell’s Na^+^ to prevent toxicity from high Na^+^ concentrations (Padan and Schuldiner, 1994).

### Genetic Constitution of CRISPRs

Clustered regularly interspaced short palindromic repeat elements are common in bacteria and archaea. A CRISPR is characterized by direct repeats (DRs) that are separated by similarly sized non-repetitive spacers, and the spacer sequences are related to antiphage functions via an RNA-silencing-like mechanism (Qin et al., 2010, 2014). CRISPRs were detected in 11 marine-derived strains and seven other habitat-origin strains (**Table 2**). The number of CRISPR locations ranges from one (in *S. oceani* 02100, *Streptomyces* sp. HPH0547, and *S. griseus* XylebKG-1) to 12 (in *S. sulphureus* L180). Seven strains of the 11 marine streptomycetes contained five or more than five CRISPRs, and the strains that were isolated from other habitats possessed less than three CRISPRs (**Table 2**). Unlike the other marine bacterium from the genus *Glaciecola* with a conservative DR in almost all the species (Qin et al., 2014), the DR length in the 18 streptomycete strains varied from 25 to 38 bp, and the DR sequences had low shared identity (Supplementary Table S7), which implies diverse origins for the CRISPRs in the *Streptomyces* genus. The number of spacers ranged from 2 to 41. The sequences of the spacers exhibited high sequence diversity. Considering the large variety of phages (approximately 5–10 times more than the quantity of bacteria) in the marine environment, it is not surprising that spacer sequences show substantial diversity (Wommack and Colwell, 2000; Qin et al., 2010).

**Table 2:** The number of CRISPRs locations and habitat of every strain.

Strains	Source	No.
*S. sulphureus* L180	Marine sediment	12
*S. sulphureus* DSM 40104	Marine	8
*Streptomyces* sp. CNB091	Marine	8
*Streptomyces* sp. CNT318	Marine	7
*Streptomyces* sp. CNH287	Marine	7
*S. nanshensis* 10372	Marine	7
*Streptomyces* sp. W007	Marine sediment	5
*Streptomyces* sp. CNS606	Marine	3
*S. nanshensis* 01066	Marine	2
*Streptomyces* sp. AA1529	Marine sediment	2
*S. oceani* 02100	Marine	1
*Streptomyces* sp. CcalMP-8W	Insect-associated	3
*Streptomyces* sp. ScaeMP-e10	Insect-associated	3
*S. griseus* NBRC 13350	Soil	3
*S. globisporus* C-1027	Soil	2
*Streptomyces* sp. Wigar10	Surface-sterilized garlic bulb	2
*Streptomyces* sp. HPH0547	*Homo sapiens*	1
*S. griseus* XylebKG-1	Ambrosia beetle *Xyleborinus saxeseni*	1

## Discussion

The marine environment is prone to high salinity and oligotrophic conditions. Therefore, it is likely that marine-derived streptomycetes would need to possess various strategies to cope with this type of environment. A phylogenetic analysis, a pan-genomics analysis and a functional genomics analysis were used to reveal the characteristics of marine-derived streptomycetes, which reflected their ability to survive in a marine environment. The phylogeny results may suggest that marine-derived streptomycetes and multiple sources have independent origins. The marine-derived streptomycete strains likely originated from a common ancestor with an ocean origin. Based on the genome dynamics analysis, we concluded that a genomic expansion in streptomycetes during the initial period help the genus adapt to different environments and divide them into subgroups. The genes that are redundant in the new environment are then lost. Finally, most genomes went through a more recent period of genome expansion along with the present day species. Furthermore, most of the genes born on terminal branches were from LGT, which affects a substantial proportion of the streptomycete pan-genome and drives genome expansion. The same pattern of genomic expansion and degradation was also observed by Cuypers and Hogeweg (2012) in a virtual cell model.

The deep sea exhibits extreme conditions, such as high hydrostatic pressures (up to 1,100 bar) and low temperatures (less than 4°C; Jorgensen and Boetius, 2007), which are challenges to marine bacterial survival. Seawater always has a high sodium concentration, which is likely to form a high-osmolality environment. The water in a cell will rapidly flux out along the osmotic gradient when microorganisms are exposed to high-osmolality environments, which may cause a reduction in turgor and the dehydration of the cytoplasm (Kempf and Bremer, 1998). In using a functional genomics analysis as a guide, we found that marine-derived streptomycete strains possessed more Trk and BCCT genes related to high-osmolality environment adaptation. We predicted that the primary strategy for marine-derived streptomycete strain survival is to accumulate K^+^ by Trk. The abundance of BCCT in marine-derived streptomycete strains may suggest that they accumulate intracellular betaine/carnitine/choline as a major adaptive response to a high-osmolality environment.

Deep-sea sediments are oligotrophic, and particulate organic matter (POM) in sediments is the major nutrient resource for deep-sea bacteria. Every year, most nutrients arrive at the deep sea in pulses, and deep-sea bacteria must therefore respond to this nutrient pulse quickly as well (Qin et al., 2011). In coping with the oligotrophic environment, marine-derived subgroup strains developed more transporters to respond to trace nutrients and absorb a variety of carbon and nitrogen sources to enhance their competitiveness. The tat family helps streptomycete strains from the marine source subgroup hydrolyze complex compounds into soluble nutrients; TRAP-T increases the absorption of nutrients, and SSS, NCS1, and NSS provide effective ways to transfer various nutrients. For most strains that were isolated from the South China Sea, there are also many other transporter families that are involved in taking up nutrients, which were already mentioned above. However, further work is needed to clarify the exact roles of these transporters in marine-derived streptomycete physiology.

The analyses presented here also confirmed the presence of genes that encode drug/metabolite transport, metalloid transport, and pH regulation. Evidently, the toxic effects of high concentrations of sugars, amino acids, other metabolites, and their detrimental analogs have forced bacteria to acquire these efflux systems during evolution (Jack et al., 2001). To ensure bacterial survival, the active efflux of drugs and metabolites is essential (Levy, 1992; Nikaido, 1994). In this study, we found that more drug or metabolite efflux transporters were processed in marine-derived streptomycete strains, such as RND, DMT, MOP, and RhtB. Many of the proteins in the DMT superfamily were found to be pumps for drug or metabolite efflux (Jack et al., 2001). Previous studies suggested that RhtB is involved in the efflux of homoserine and threonine from *Escherichia coli* (Aleshin et al., 1999), and it also might be involved in quorum sensing (Vicente et al., 2009). These drug or metabolite pumps may help marine-derived streptomycete strains eject intracellular toxic substances, and they are beneficial for survival in the marine environment. In addition, the abundance of CRISPRs in marine isolates may also help marine streptomycetes against phages in the marine environment.

In summary, by comparing the genomes of ocean isolates with those of isolates from other environments, we are beginning to identify some genomic characteristics that enable marine streptomycetes to thrive in complex marine environments. Phylogenetic analyses have revealed the dynamic process of streptomycete genome evolution, and LGT is an important driving force during this process, as already shown by Ochman et al. (2000). Pan-genomic analyses revealed that streptomycetes have an open pan-genome that reflects the diversity of these streptomycete species and their high levels of adaptability to different environments. The functional genomics analyses indicate that marine-derived subgroup streptomycetes possess some characteristics of marine adaptation, such as high hydrostatic pressure, low temperature, and oligotrophic adaptability. To adapt to the high hydrostatic pressure and low temperature conditions, marine-derived subgroup strains accumulate more functional genes that are related to post-translational modification, protein turnover, chaperones, translation, ribosomal structure and biogenesis as well as more BCCT, Trk, MscS, and MscL transporter families. The genomes of most marine-derived subgroup strains harbored a variety of transporters to cope with the oligotrophic environment, and these transporters included TRAP-T, SSS, NCS1, NSS, Tat, TTT, GntP, DAACS, LctP, POT, GPTS, and Amt. Furthermore, more transporters take part in drug/metabolite efflux, metalloid resistance, and pH regulation, thereby adapting to other marine conditions, and they are contained in marine-derived subgroup strains. In sum, marine streptomycetes possess more functional genes and transporters than other streptomycetes to adapt to the cold, hyperosmosis, oligotrophy, and other marine conditions, and marine isolates also have more CRISPRs, which is related to phage resistance.

### Data Accessibility

The nine draft genome sequences of *Streptomyces* were submitted to the NCBI. The accession numbers were JHEJ00000000, LJGS00000000, LJGT00000000, LJGU00000000, LJGV00000000, LJGW00000000, LJGX00000000, LJGY00000000, and LJGZ00000000.

## Author Contributions

XT, Zhewen Zhang, and TY contributed equally to this manuscript. MC, FC, Zhang Zhang, JW, CZ, LL, and JX participated in the design and discussion of the research. XT, JL, JY, and BZ carried out the experimental part of the work. Zhewen Zhang and TY carried out the analysis of the data and wrote the manuscript. All authors have read and approved the final manuscript.

## Conflict of Interest Statement

The authors declare that the research was conducted in the absence of any commercial or financial relationships that could be construed as a potential conflict of interest.

## Footnotes

1 http://www.genome.jp/kaas-bin/kaas_main
