# Supplementary material for: Comparative Genomics Analysis of Streptomyces Species Reveals Their Adaptation to the Marine Environment and Their Diversity at the Genomic Level
Source: Front Microbiol. 2016 Jun 27;7:998. doi: 10.3389/fmicb.2016.00998 (PMC4921485; doi:10.3389/fmicb.2016.00998)
Supplement: Supplementary file 1 [file Table_1.PDF]

Comparative genomics analysis of *Streptomyces* species reveals their adaptation to the marine environment and their diversity at the genomic level

Xinpeng Tian[1#], Zhewen Zhang[2#], Tingting Yang[2,3#], Meili Chen[2], Jie Li[1], Fei Chen[2], Jin Yang[4], Wenjie Li[4], Bing Zhang[4], Zhang Zhang[2], Jiayan Wu[2], Changsheng Zhang[1], Lijuan Long [1]*, Jingfa Xiao[2]*

[*] Corresponding author.

E-mail address: longlj@scsio.ac.cn (LJ Long); xiaojingfa@big.ac.cn (JF XIAO).

Supplementary Tables

Table S1. Habitat information of 9 Streptomyces strains isolated from the South China Sea area.

Strain No.	Species name	Sea area	Longitude	Latitude	Depth （m）
10389	*Streptomyces abyssalis*	Sanya	Gorgonian*		
10390	*Streptomyces abyssalis*	Sanya	Gorgonian*		
02100	*Streptomycetes oceani*	South China Sea	116°36.100′E	20°14.189′N	578
10379	*Streptomyces qinglanensis*	South China Sea	114°34′16.668″E	17°59′48.135″	3,587
10429	*Streptomyces nanshensis*	Sanya	Gorgonian*		
01066	*Streptomyces nanshensis*	Nansha, South China Sea	113° 33′E	6°40′N	2,015
10399	*Streptomyces nanshensis*	South China Sea	110°20.520′	17°12.120′	1,366
10374	*Streptomyces nanshensis*	South China Sea	110°28′46.641″E	17°27′34.988″	1,198
10372	*Streptomyces nanshensis*	South China Sea	110°28′46.641″E	17°27′34.988″	1,198

*Note: the gorgonians were not identified.

Table S2.Basic information of the 22 strains downloaded from NCBI.

**Strains**	**Source**	**Status**	**Accession number**	**Geographic Location**
S.sulphureus DSM 40104	Marine	Draft	ARLC00000000	
Streptomyces sp. CNB091	Marine	Draft	ARJI00000000	
Streptomyces sp. CNT318	Marine	Draft	AUKN00000000	
Streptomyces sp. CNT360	Marine	Draft	AUKO00000000	
Streptomyces sp. CNH287	Marine	Draft	AUKQ00000000	
Streptomyces sp. TAA204	Marine	Draft	AUKW00000000	
Streptomyces sp. TAA486	Marine	Draft	AUEV00000000	
Streptomyces sp. CNS606	Marine	Draft	AUFD00000000	
S.sulphureus L180	Marine sediment	Draft	AJTQ00000000	Dalian,China*
Streptomyces sp. W007	Marine sediment	Draft	AGSW00000000	Jiaozhou Bay, China
Streptomyces sp. AA1529	Marine sediment	Draft	ALAP00000000	yellow sea, China
S.globisporus C-1027	Soil	Draft	AJUO00000000	Hubei, China*
S.griseus NBRC 13350	Soil	Finish	AP009493	
S.roseosporus NRRL 11379	Soil	Draft	ABYB00000000	
S.roseosporus NRRL 15998	Soil	Draft	ABYX00000000	
Streptomyces sp. CcalMP-8W	Insect-associated	Draft	ARDQ00000000	
Streptomyces sp. ScaeMP-e10	Insect-associated	Draft	ARDV00000000	
S.griseus XylebKG-1	Ambrosia beetle Xyleborinus saxeseni	Draft	ADFC00000000	
Streptomyces sp. Wigar10	Surface-sterilized garlic bulb	Draft	AGDF00000000	Columbus, USA
Streptomyces sp. HPH0547	Homo sapiens	Draft	ATCE00000000	Digestive system
S.fulvissimus DSM 40593	Unknown	Finish	CP005080	
Streptomyces sp. HCCB10043	Unknown	Draft	AWOQ00000000	

*Note：Streptomyces sp. W007 was isolated from longitude E120°14'44.16", latitude N36°7'24.456", and depth 50 m; And S.globisporus C-1027 was isolated from longitude E112°54'2.16", latitude N30°24'33.48", and depth 10 m.

Table S3. Total reads length (after quality filter) of each library, genome size, and average genome coverage of each strain isolated from South China Sea.

Strains	500bp (bp)	1-3kb (bp)	3-5kb (bp)	Total (bp)	Genome size (bp)	Coverage
S. oceani 02100	1,139,919,630	2,101,370,624	630,644,578	3,871,934,832	6,333,177	611.37
S. nanshensis 01066	1,313,470,341	734,126,291	731,765,943	2,779,362,575	7,431,922	373.98
S. nanshensis 10372	2,017,799,978	-	927,807,231	2,945,607,209	10,271,727	286.77
S. nanshensis 10374	2,326,774,128	1,802,181,799	1,023,478,150	5,152,434,077	6,471,789	796.14
S.qinglanensis 10379	1,624,461,814	1,463,486,739	1,171,815,095	4,259,763,648	6,610,646	644.38
S. abyssalis 10389	1,317,640,515	948,530,130	1,188,093,044	3,454,263,689	6,910,240	499.88
S. abyssalis 10390	2,027,583,208	1,394,727,205	1,522,639,493	4,944,949,906	6,610,640	748.03
S. nanshensis 10399	1,103,483,328	-	1,465,189,886	2,568,673,214	8,576,685	299.49
S. nanshensis 10429	1,305,739,508	800,863,458	525,428,896	2,632,031,862	7,725,085	340.71

Table S4. The result of genome assembly of 9 marine *Streptomyces*.

**Strains**	**Scaffold**				**Contig**			
	**No.**	**total size （bp）**	**N50 （bp）**	**N90 （bp）**	**No.**	**total size （bp）**	**N50 （bp）**	**N90 （bp）**
S. oceani 02100	104	6,333,177	1,028,693	151,422	349	6,240,700	49,837	11,138
S. nanshensis 01066	2,310	7,431,922	14,442	1,335	3,061	7,349,464	8,695	1,057
S. nanshensis 10372	114	10,271,727	349,090	77,932	1,257	9,771,120	13,457	3,489
S. nanshensis 10374	50	6,471,789	4,098,964	881,363	116	6,442,749	193,769	62,553
S.qinglanensis 10379	22	6,610,646	5,718,470	870,851	72	6,595,063	440,771	88,701
S. abyssalis 10389	45	6,910,240	775,892	306,638	284	6,704,172	58,385	13,075
S. abyssalis 10390	42	6,610,640	1,628,582	556,846	58	6,599,740	652,893	133,775
S. nanshensis 10399	1,285	8,576,685	29,825	3,203	2,699	6,910,744	4,130	1,260
S. nanshensis 10429	731	7,725,085	19,860	5,267	1,024	7,694,665	14,924	4,059

Table S5 Relative effect of recombination and mutation on each strain.

**Strains**	**R/theta**[a]	**delta**[b]	**nu**[c]	**r/m**[d]
**S. nanshensis* 01066	0.07	54.12	0.25	0.93
**S. oceani* 02100	4.01	215.80	0.13	116.45
**S. nanshensis* 10372	0.14	43.56	0.08	0.51
**S. nanshensis* 10374	0.75	95.35	0.04	2.69
**S. qinglanensis* 10379	0.62	107.04	0.04	2.39
**S. abyssalis* 10389	0.63	97.82	0.04	2.16
**S. abyssalis* 10390	0.58	104.80	0.04	2.14
**S. nanshensis* 10399	0.85	90.66	0.12	8.89
**S. nanshensis* 10429	1.59	129.39	0.08	16.95
**Streptomyces* sp. AA1529	0.05	49.37	0.19	0.45
S. griseus XylebKG-1	0.04	64.86	0.08	0.23
Streptomyces sp. CcalMP-8W	1.24	122.80	0.02	3.52
**Streptomyces* sp. CNB091	0.51	51.73	0.05	1.44
**Streptomyces* sp. CNH287	2.47	121.37	0.10	29.05
**Streptomyces* sp. CNS606	2.63	128.43	0.13	42.76
**Streptomyces* sp. CNT318	0.43	73.82	0.01	0.43
**Streptomyces* sp. CNT360	1.38	117.35	0.09	14.07
S. fulvissimus DSM 40593	1.44	75.77	0.05	4.91
S. globisporus C-1027	0.56	55.15	0.05	1.49
Streptomyces sp. HCCB10043	0.76	92.77	0.20	14.22
Streptomyces sp. HPH0547	1.22	126.30	0.08	12.66
**S. sulphureus* L180	2.36	492.06	0.01	16.33
S. griseus NBRC 13350	0.05	54.04	0.15	0.41
S. roseosporus NRRL 11379	0.89	87.12	0.16	12.71
S. roseosporus NRRL 15998	0.24	88.01	0.34	7.15
Streptomyces sp. ScaeMP-e10	1.49	87.80	0.04	5.36
**S. sulphureus* DSM 40104	2.12	376.30	0.02	15.90
**Streptomyces* sp. TAA204	1.62	148.18	0.09	21.54
**Streptomyces* sp. TAA486	1.53	203.44	0.07	20.72
**Streptomyces* sp. W007	0.46	66.08	0.05	1.52
Streptomyces sp. Wigar10	0.20	24.97	0.44	2.18

Marine isolated strains were marked by asterisk.

a. the ratio of rates of recombination and mutation

b. the average length of recombined fragments

c. average divergence between donor and recipient

d. the ratio of effects of recombination and mutation

Table S6 The number of transporters according to TransportDB transporter family in 31 *Streptomyces*.

Family	1	2	3	4	5	6	7	8	9	10	11	12	13	14	15	16	17	18	19	20	21	22	23	24	25	26	27	28	29	30	31
SSS	3	2	4	3	2	2	2	3	2	7	6	6	2	0	3	0	0	1	1	1	1	1	1	1	0	1	1	2	1	1	1
Nramp	3	2	1	1	1	1	1	1	1	5	4	4	5	2	3	1	2	4	1	1	1	2	2	1	1	1	1	2	2	1	1
TRAP-T	1	2	1	1	1	1	1	1	1	4	4	4	4	4	4	1	1	1	1	1	1	1	1	1	1	1	1	1	1	1	1
BCCT	4	4	1	2	2	3	3	3	3	3	1	1	1	1	2	1	2	2	1	1	1	1	2	2	2	2	1	1	2	1	1
Trk	1	1	1	1	1	2	2	3	3	1	1	1	1	1	3	1	3	3	1	1	1	1	1	1	1	1	1	1	1	1	1
Tat	2	2	1	1	5	5	5	2	2	3	3	3	1	1	1	2	2	2	0	0	0	0	0	0	0	0	0	0	0	0	1
MOP	3	3	1	1	2	4	4	1	1	5	3	3	1	0	2	2	0	3	1	1	1	0	0	0	1	0	2	5	1	0	0
NSS	1	1	0	1	1	1	1	1	1	1	0	0	0	0	0	1	1	1	1	1	1	0	0	0	0	1	0	0	0	0	0
RhtB	0	1	1	2	3	2	2	1	1	1	1	1	0	0	1	0	1	0	0	0	0	0	0	0	0	0	0	1	1	0	0
ArsB	0	0	0	0	0	1	1	0	0	1	1	1	1	0	0	0	0	0	0	0	0	0	0	0	0	0	1	1	0	0	0
ThrE	1	1	0	0	1	1	1	0	0	1	1	1	0	0	0	0	0	0	0	0	0	0	0	0	0	0	1	0	0	0	0
LIV-E	0	0	0	0	1	1	1	0	0	1	1	1	0	0	0	0	0	0	0	0	0	0	0	0	0	0	0	1	0	0	0
DAACS	0	0	0	0	1	1	1	0	0	1	1	1	0	0	0	0	0	0	0	0	0	0	0	0	0	0	0	1	0	0	0
LctP	0	0	0	0	1	1	1	0	0	2	1	1	0	0	0	0	0	0	0	0	0	0	0	0	0	0	0	0	0	0	0
MscL	0	0	0	0	2	1	1	0	0	1	1	1	0	0	0	0	0	0	0	0	0	0	0	0	0	0	0	0	0	0	0
Hsp70	0	0	0	0	1	1	1	0	0	1	1	1	0	0	0	0	0	0	0	0	0	0	0	0	0	0	0	0	0	0	1
GPTS	0	1	0	0	2	2	1	0	0	2	2	2	0	0	0	0	0	0	0	0	0	1	0	0	0	0	2	1	0	0	1
PiT	0	0	0	0	3	2	2	0	0	1	2	2	0	0	0	0	0	0	0	0	0	0	0	0	0	0	0	2	0	0	0
ACR3	0	0	0	0	1	1	1	0	0	1	1	1	0	0	0	0	0	0	0	0	0	0	0	0	0	0	0	4	0	0	0
NhaA	0	0	0	0	3	3	3	0	0	2	0	0	0	0	0	1	0	0	0	0	0	0	0	0	0	0	0	3	0	0	0
DASS	0	0	1	0	0	0	0	0	0	0	0	0	2	3	1	1	1	0	1	1	1	1	1	1	1	1	0	0	0	1	1
AAE	0	0	0	0	0	0	0	0	0	0	0	0	0	0	0	0	0	0	1	1	0	1	2	2	2	2	1	2	1	1	2
MerTP	0	1	0	0	2	2	2	0	0	0	0	0	0	0	0	1	0	0	1	1	1	2	1	1	0	1	1	1	2	2	1

NCS1	0	1	6	5	4	2	2	2	2	3	1	1	1	0	0	0	0	0	0	0	0	1	1	0	0	0	1	0	0	0	0
POT	0	0	0	0	2	2	2	0	0	3	1	1	0	0	0	0	0	0	0	0	0	0	0	0	0	0	0	1	0	0	0
MscS	0	0	0	0	3	3	2	0	0	4	2	2	0	0	0	0	0	0	0	0	0	0	0	0	1	1	0	0	0	0	0
Amt	0	0	0	0	3	3	3	0	0	2	2	2	0	0	0	0	0	0	0	0	0	0	0	0	0	0	0	0	0	0	0
TTT	0	0	1	1	4	2	2	0	0	5	2	3	1	1	1	1	1	2	0	0	0	0	0	0	0	0	0	2	0	0	0
GntP	0	0	0	0	2	2	2	0	0	5	3	3	0	0	0	0	0	0	0	0	0	0	0	0	0	0	0	2	0	0	0
MIP	0	0	0	0	3	3	3	0	0	5	2	2	0	0	0	0	0	0	0	0	0	0	0	0	0	0	1	3	0	0	0
NCS2	0	0	0	0	4	3	3	0	0	5	3	3	0	0	0	0	0	0	0	0	0	0	0	0	0	0	1	3	0	0	0

Note: numbers in the header on behalf of the name of strains. 1, *S.sulphureus* DSM 40104; 2, *S.sulphureus* L180; 3, *Streptomyces* sp. CNT360; 4, *Streptomyces* sp. HPH0547; 5, *S.nanshensis* 10399; 6, *S.qinglanensis* 10379; 7, *S.nanshensis* 10374; 8, *Streptomyces* sp. AA1529; 9, *Streptomyces* sp. CNT318; 10, *S.nanshensis* 01066; 11, *S.abyssalis* 10389; 12, *S.abyssalis* 10390; 13, *Streptomyces* sp. TAA486; 14, *S.nanshensis* 10429; 15, *Streptomyces* sp. TAA204; 16, *Streptomyces* sp. CNH287; 17, *S.oceani* 02100; 18, *Streptomyces* sp. CNS606; 19, *S.roseosporus* NRRL 15998; 20, *S.roseosporus* NRRL 11379; 21, *Streptomyces* sp. HCCB10043; 22, *S.globisporus* C-1027; 23, *S.griseus* NBRC 13350; 24, *S.griseus* XylebKG-1; 25, *Streptomyces* sp. W007; 26, *Streptomyces* sp. CNB091; 27, *Streptomyces* sp. Wigar10； 28, *S.nanshensis* 10372; 29, *Streptomyces* sp. CcalMP-8W; 30, *S.fulvissimus* DSM 40593; 31, *Streptomyces* sp. ScaeMP-e10.

Table S7. Information of CRISPRs in *Streptomyces*.

Strains	No.	DR length (bp)	Spacer length (bp)	Spacer No.	Sequence of DR
S.nanshensis 01066	2	30	35-38	5	GTTGCTACCCCTCGCAGGGGCGATGAGGAC
		29	37-38	3	GTTGCTACCCCTCGCAGGGGCGATGAGGA-
S.oceani 02100	1	29	32	4	GGGGCCAACCCCGCGTGCGCGGGGAGCAC
S.nanshensis 10372	7	29	32	3	GGGAACACCCCCGCGTGCGCGGGGA-----CCAC
		29	32-40	8	---GTGGTCCCCGCGCGTGCGGGGGTGGTCCC--
		26	32-35	10	-----GGTCCCCGCGCGTGCGGGGGTGGTCC---
		30	31	3	---GTGGTCCCCGCGCGAGCGGGGGTGTTCCGG-
		32	29	2	--AGTGGTCCCCGCGCGAGCGGGGCTGTTCCGTC
		29	32-42	3	---GTGGTCCCCGCGCACGCGGGGATGGTCCC--
		30	31	4	---GTGGTCCCCGCGCACGCGGGGATGGTCCCG-
S.sulphureus L180	12	29	30-53	11	---CTGCTCCCCGCGCACGCGGGGATGGACCC-
		29	31-32	10	---CTGCTCCCCGCGCACGCGGGGATGGACCC-
		28	33-34	4	---GTGGTCCCCGCGCACGCGGGGGTGGTCC--
		28	33	8	---GTGGTCCCCGCGCACGCGGGGGTGGTCC--
		29	32	6	---GTGCTCCCCGCGCGTGCGGGGGTGGTCCC-
		32	29	2	-GAGTGGTCCCCGCGCGTGCGGGGGTGGTCCGG
		29	32	7	GGGTCCATCCCCGCACGCGCGGGGA----GCAG
		29	32	9	---CTGCTCCCCGCGCACGCGGGGATGGACCC-
		30	31	4	---CTGCTCCCCGCGCGTGCGGGGATGGTCCCC
		29	32	4	---CTGCTCCCCGCGCGTGCGGGGATGGTCCC-
		29	32-34	15	---CTGCTCCCCGCGCACGCGGGGATGGACCC-
		29	32	13	---GTGCTCCCCGCGCGTGCGGGGGTGGTCCC-
S.sulphureus DSM 40104	8	29	32	4	-GGGACCACCCCCGCACGCGCGGGGAGCAC
		28	33	9	--GGTCCATCCCCGCGTGCGCGGGAAGCAG
		29	32-33	14	-GGGTCCATCCCCGCGTGCGCGGGGAGCAG
		29	32	5	-GGGACCATCCCCGCACGCGCGGGGAGCAG
		30	31	4	CGGGTCCATCCCCGCGCACGCGGGGAGCAG
		29	32	11	-GGGTCCATCCCCGCACGCGCGGGGAGCAG
		28	33	4	GTGGTCC--CCGCGCATGCGGGGGTGGTCC
		27	34-35	6	GTGCTCC--CCGCGCGTGCGGGGGTGGTC-
Streptomyces sp. AA1529	2	29	31	4	ACTGCTCCCCGCGCAGCGGGGATGGACCC
		28	32	4	-CTGCTCCCCGCGCAGCGGGGATGGACCC
Streptomyces	7	28	33-34	9	--GGAGCATCCCCGCGGGCGCGGGGTCGAC---

sp. CNT318					
		29	30-32	13	-GGGACCATCCCCGCGGGCGCGGGGAGCAC---
		28	33	7	GTGC----TCCCCGCGCGAGCGGGGATGGTCC-
		28	33	11	GTGG----TCCCCGCGCGAGCGGGGGTGGTCC-
		28	33-34	4	----GTCGACCCCGCGCCCGCGGGGATGCTCC-
		28	31-34	7	----GTCGACCCCGCGCCCGCGGGGATGCTCC-
		29	32-33	3	GTGC----TCCCCGCGCGAGCGGGGATGGTCCC
Streptomyces sp. HPH0547	1	28	33	4	GGACCAACCCCGCGCGTGCGGGGAGCAC
Streptomyces sp. CNS606	3	30	33	2	CGGGACCATCCCCGCGGGCGCGGGGAGCAG
		29	31	3	-GGGACCATCCCCGCGGGTGCGGGGAGCAG
		28	32	4	GATCGCTCCGTGCGCCCGTGGGCCGGAC
Streptomyces sp. CNH287	7	29	32	2	CCTGCTCCCCGCGCACGCGGGGATGGACC-
		29	32	4	-CTGCTCCCCGCGCACGCGGGGATGGACGC
		30	32	2	TCTGCTCCCCGCGCACGCGGGGATGGACCC
		29	32	12	-CTGCTCCCCGCGCACGCGGGGATGGACCC
		29	31	6	-CTGCTCCCCGCGCACGCGGGGATGGACCC
		28	33-37	6	-CTGCTCCCCGCGCACGCGGGGATGGACC-
		27	37-46	7	TGCGAACCCACCAGGGTTGATGACGGC
Streptomyces sp. Wigar10	2	29	32	2	GTGGTCCCCGCGCGAGCGGGGGTGTTCCG
		29	32	3	GTGGTCCCCGCGCACGCGGGGGTGTTCCC
Streptomyces sp. CcalMP-8W	3	29	32	3	CGGAACACCCCCGCTCGCGCGGGGA---CCAC
		29	32	6	GGGAACACCCCCGCGTGCGCGGGGA---CCAC
		29	32	4	---GTGGTCCCCGCGCGTGCGGGGATGGTCCC
Streptomyces sp. ScaeMP-e10	3	29	31-36	41	GTGGTCCCCGCGCAGGCGGGGGTGTTCCG
		29	32	4	GTGGTCCCCGCGCACGCGGGGGTGGTCCC
		28	33-38	4	GTGGTCCCCGCGCAGGCGGGGGTGTTCC-
S.globisporus C-1027	2	35	35-39	4	---GGCGGTCGCCCTCCGGGGTGGCCGAGGATCGCAAC
		38	35-37	3	TGTGGCGGTCGCCCTCCGGGGTGGCCGAGGATCGCAAC
S.griseus NBRC 13350	3	29	32	16	GTGGTCCCCGCGCGTGCGGGGGTGTTCCC
		29	32	6	GTGGTCCCCGCGCGTGCGGGGGTGTTCCC
		28	32-33	17	GTGGTCCCCGCGCGTGCGGGGTTGTTCC-
S.griseus XylebKG-1	1	29	32	2	CGGCTCACCTCCGCTCGCGCGGAGAGCAC

Streptomyces sp. W007	5	29	32	3	--CGGCTCACCTCCGCTCGCGCGGA---GAGCAC
		29	32-37	6	--CGGCTCACCTCCGCTCGCGCGGA---GAGCAC
		31	30	3	AGCGGCTCACCTCCGCTCGCGCGGA---GAGCAC
		29	32	4	--GTGCT---CTCCGCGCGAGCGGAGGTGAGCCG
		29	32	4	--CGGCTCACCTCCGCTCGCGCGGA---GAGCAC
Streptomyces sp. CNB091	8	25	49-56	3	TCTGTCCTCAAGCGCCGGACGGGCT
		29	31-41	25	--GGGACCAACCCCGCACGCGCGGGGACGAC--
		31	30	4	CCGGGACCACCCCCGCGTGCGCGGGGACCAC--
		29	32	6	--GGGACCACCCCCGCACGCGCGGGGACCAC--
		28	33	6	ATGG-----TCCCCGCGCACGCGGGGGTGGTCC
		28	33	3	-CGGGACCACCCCCGCGTGCGCGGGGACC----
		29	32	4	--GGGACCATCCCCGCGTGCGCGGGGACCAC--
		30	31	2	-GGCAACCACCCCCGCGTGCGCGGGGACCGT--
