# Supplementary material for: Comparative Genomics Analysis of Streptomyces Species Reveals Their Adaptation to the Marine Environment and Their Diversity at the Genomic Level
Source: Front Microbiol. 2016 Jun 27;7:998. doi: 10.3389/fmicb.2016.00998 (PMC4921485; doi:10.3389/fmicb.2016.00998)
Supplement: Supplementary file 2 [file Image_1.PDF]

Comparative genomics analysis of *Streptomyces* species reveals their adaptation to the marine environment and their diversity at the genomic level

Xinpeng Tian[1#], Zhewen Zhang[2#], Tingting Yang[2,3#], Meili Chen[2], Jie Li[1], Fei Chen[2], Jin Yang[4], Wenjie Li[4], Bing Zhang[4], Zhang Zhang[2], Jiayan Wu[2], Changsheng Zhang[1], Lijuan Long [1]*, Jingfa Xiao[2]*

[*] Corresponding author.

E-mail address: longlj@scsio.ac.cn (LJ Long); xiaojingfa@big.ac.cn (JF XIAO).

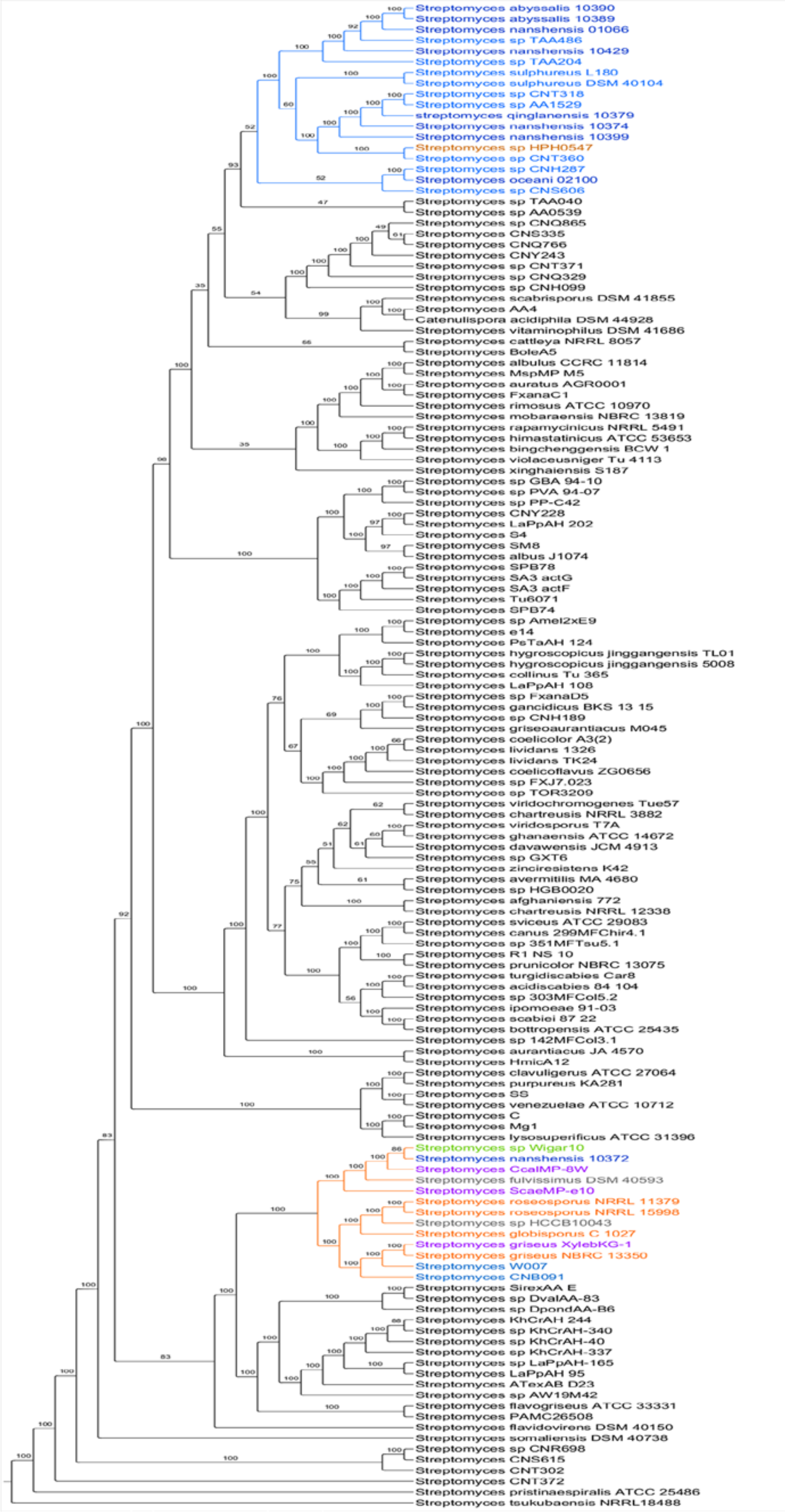

Figure S1. Phylogenetic tree of 136 streptomycete strains constructed on 136 streptomycete genomes by Co-phylog method.

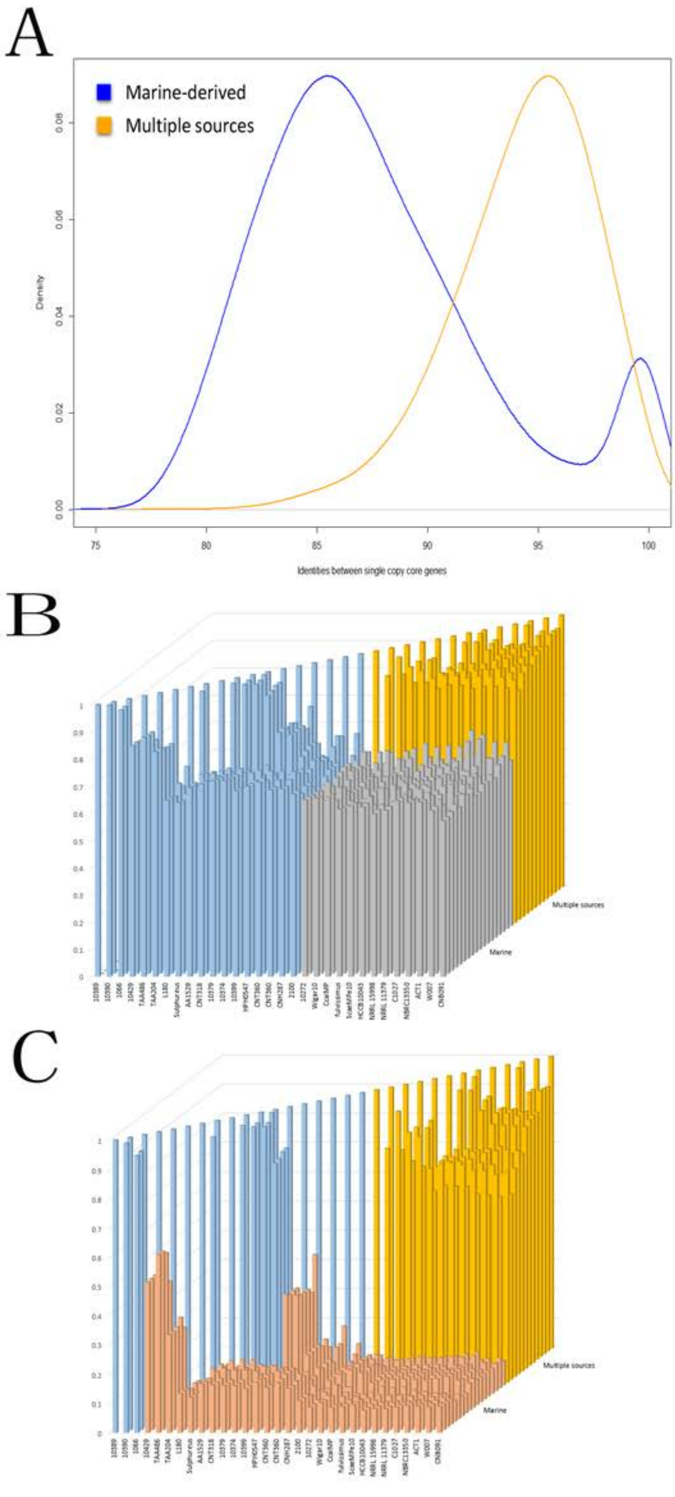

Figure S2. The identity distribution between single copy core genes. **(A)** The identity density of pairwise single copy core genes. **(B)** The percentages of genes with 80% or higher shared identity among 31 streptomycete strains. **(C)** The percentages of genes with more than 90% shared identity among 31 streptomycete strains. Blue indicates the marine-derived subgroup and yellow indicates the multiple sources subgroup.
